# Fluorodeoxyglucose Positron Emission Tomography/Computed Tomography Imaging of a Patient with Squamous Cell Carcinoma of Prostate

**DOI:** 10.1155/2014/860570

**Published:** 2014-03-24

**Authors:** Gonca Kara Gedik, Guler Yavas, Murat Akand, Esin Celik, Oktay Sari

**Affiliations:** ^1^Department of Nuclear Medicine, Faculty of Medicine, Selcuk University, Selcuklu, Konya, Turkey; ^2^Department of Radiation Oncology, Faculty of Medicine, Selcuk University, Selcuklu, Konya, Turkey; ^3^Department of Urology, Faculty of Medicine, Selcuk University, Selcuklu, Konya, Turkey; ^4^Department of Pathology, Faculty of Medicine, Selcuk University, Selcuklu, Konya, Turkey

## Abstract

Primary squamous cell carcinoma is an uncommon tumor of the prostate gland. We report a 77-year-old male patient with urinary frequency and constipation. Fine needle biopsy from prostate was suspicious of squamous cell carcinoma of the prostate. Whole body positron emission tomography/computed tomography scan revealed high fluorodeoxyglucose uptake in prostate gland. Transurethral resection confirmed the diagnosis. In contrast to prostatic adenocarcinoma, high fluorodeoxyglucose accumulation was observed in the primary tumor of the prostate gland.

## 1. Introduction

Primary squamous cell carcinoma of the prostate is a rare neoplasm of prostate gland and comprises 0.5–1% of all prostatic carcinomas [1]. It differs from common prostatic adenocarcinoma in its therapeutic response and prognosis. This type of prostate carcinoma has a poor response to conventional treatment resulting from poor prognosis [1].

The initial diagnostic procedure for a patient with suspected prostate cancer is multiple site blind prostate biopsies. Among currently available nuclear medicine studies, positron emission tomography (PET) has some potential to detect primary tumor in prostate cancer. In prostatic adenocarcinoma, however, fluorodeoxyglucose (FDG) is inferior to other PET tracers like choline and acetate and conducted studies using FDG PET in localized prostate cancer reported disappointing results [2].

Herein, we report a patient with squamous cell carcinoma of prostate, in whom high FDG accumulation was observed in primary tumor site. The difference of FDG affinity between prostatic adenocarcinoma and squamous cell cancer of prostate is discussed and the literature is reviewed.

## 2. Case Report

**Figure 1:**
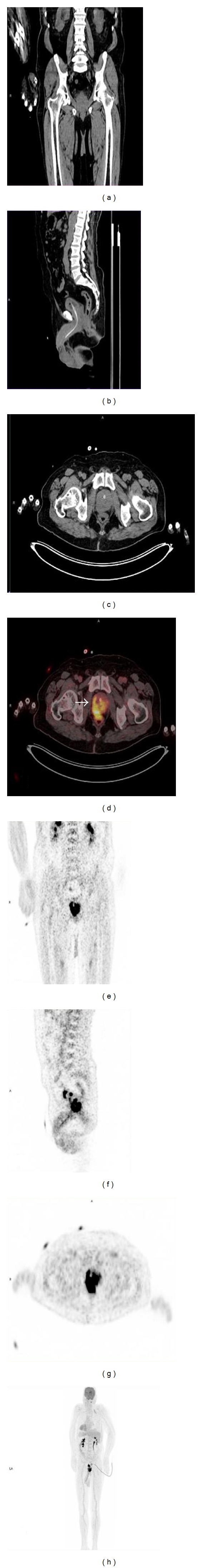
Coronal (a), sagittal (b), transaxial (c) CT, fused PET/CT (d) and coronal (e), sagittal (f), transaxial (g) PET, and MIP (h) images of the patient. Increased FDG uptake extending to the rectal wall was observed in the posterolateral part of the prostate gland ((d), arrow).

**Figure 2:**
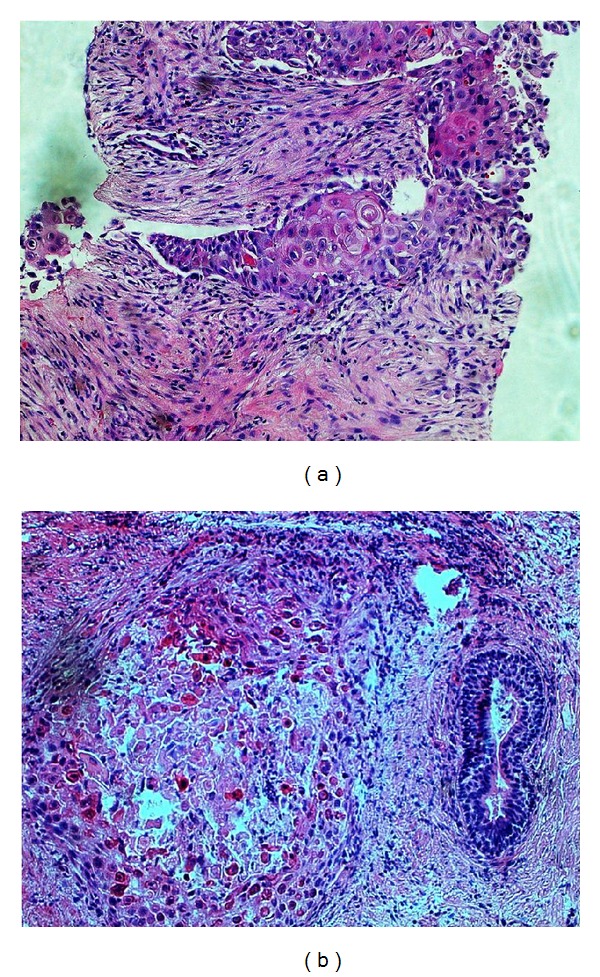
(a) Tumor cells with atypical, pleomorphic nuclei, prominent nucleoli, and cytoplasmic keratotic features infiltrating prostatic stroma (HEx200). (b) Tumor island with central necrosis is seen near a prostatic duct (HEx200).

A 77-year-old man admitted to our hospital with complains of constipation and hesitancy. He had decreased urinary output and severe constipation for 5-6 months. Clinical examination revealed an enlarged and firm prostate. Laboratory evaluation showed that serum prostate specific antigen (PSA) levels were in normal levels (4.3 ng/mL, normal: 0–4.5 ng/mL). However, serum creatinine and urea levels were 2.42 mg/dL (normal: 0.4–1 mg/dL) and 87.5 mg/dL (normal: 11–36 mg/dL), respectively. The uroflowmetry test was compatible with an obstructive pathology. Since the patient has severe constipation, a rectosigmoidoscopy and fine needle biopsy were performed which was suspicious for squamous cell carcinoma of the prostate. Fluorodeoxyglucose positron emission tomography computed tomography (FDG PET/CT) was performed for staging. Dual modality PET/CT examination was obtained after intravenous administration of 370 megabecquerel FDG with using an integrated scanner (Biograph mCT, Siemens). The PET scan was started immediately after unenhanced CT. PET/CT examination revealed an increased 18F-FDG uptake in the prostate expanding through the rectum with a maximum standardized uptake value (SUVmax) of 29.73 (Figure 1). Increased 18F-FDG uptake was also observed in the right parailiac lymph nodes (SUVmax: 11.40). After FDG PET/CT scan, transurethral resection of prostate was performed. On histopathologic examination, a solid tumor infiltrating fibromuscular prostate stroma was seen. Tumor cells with atypical, pleomorphic nuclei, prominent nucleoli, and keratotic cytoplasm constituted solid sheets with some central foci of necrosis (Figures 2(a)-2(b)). Immunohistochemically, tumor cells were positive for CK5/6, weak positive for p63, and negative for PSA. Tumor was diagnosed as “squamous cell carcinoma of prostate” with histopathologic and immunohistochemical findings.

The optimal treatment of the primary squamous cell carcinoma is still unknown. We planned to start with pelvic radiotherapy. The radiotherapy dose was planned as 50.4 Gray (Gy) to the pelvic region with pelvis box technique, a 16 Gy boost dose to the prostate gland, and a 10 Gy boost dose to the metastatic lymph nodes. However, at 6th day of radiotherapy the patient was admitted to the emergency service with a complaint of severe nausea and vomiting. Laboratory work-up revealed a creatinine level of 3, 35 mg/dL and serum urea levels of 248 mg/dL with metabolic acidosis, thought to be secondary to acute kidney injury. The patient consulted the nephrology department for dialyses; he could not continue to the radiotherapy until his metabolic acidosis and vomiting got better. Therefore we gave a break to radiotherapy for a week, and then he continued with his treatment.

## 3. Discussion

The initial diagnostic procedure for a patient with suspected prostate cancer is multiple site blind prostate biopsies [2]. However, prostate biopsy suffers from sampling error so a diagnostic test which may play a role in guiding biopsy is needed to prevent false negative results in patients with prostate carcinoma.

Squamous cell carcinoma of prostate is extremely rare clinical and pathological entity. It usually presents with lower urinary tract symptoms or symptoms related to bony metastases. In terms of clinical markers, in contrast to ordinary prostate adenocarcinoma, the squamous variant does not result in elevated levels of prostatic acid phosphatase (PAP) or PSA [3] and has a worse prognosis. Like its natural history, FDG affinity of squamous cell carcinoma of prostate is also different from prostatic adenocarcinoma. For prostatic adenocarcinoma FDG is not effective for primary diagnosis but promising results have been shown by 11C-choline, flourocholine, 11C-acetate, and 18F-fluoride [2]. Slow metabolic rate and fewer number of Glut-1 binding sites have been accused for the lower FDG uptake of this kind of prostate cancer. FDG, however, has been reported to have a role in recurrence detection in prostatic adenocarcinoma [2].

To date, few cases with primary squamous cell carcinoma of the prostate have been reported in the literature [3]. However, FDG PET/CT imaging was performed only in 2 of them. Malik et al. [3] demonstrated extensive FDG uptake throughout the prostate gland in a patient with squamous cell carcinoma of prostate; however SUVmax value was not noted. Similarly, strong FDG uptake of a lesion in prostate with SUVmax of 15.50 was reported by Dong et al in another patient with squamous cell carcinoma of prostate [4]. In our patient, high FDG uptake in prostate gland and also in right parailiac lymph nodes was observed in PET/CT scan with SUVmax values of 29.73 and 11.40, respectively. No bone metastases were noted. The patient started to receive radiotherapy but we had a week of treatment break at 6th day of radiotherapy because of severe nausea and vomiting which was a result of acute renal failure. We think that this condition might be explained as an aggressive disease which did not respond to the radiotherapy. With normal serum PSA levels and the aggressive behaviour, the presentation and the clinical course of our patient were similar to the ones reported in the literature.

Histogenesis of squamous cell carcinoma remains unclear. Some authors suggest that the prostatic urethral urothelium is the origin of squamous cell carcinoma [5]. Others believe it arises from the transitional epithelium of periurethral ducts or the basal cells of prostatic acini [5]. Taking together the difference of origin between adenocarcinoma and squamous cell carcinoma and aggressive clinical behaviour of the latter one, we thought that increased cell proliferation in this kind of tumors results in increased glucose utilization and higher Glut-1 binding site expression. This results in high FDG uptake in the primary tumor and its metastatic sites.

In prostatic adenocarcinoma, primary cancer shows relatively low FDG uptake and the same is true for nodal metastases. Limited sensitivity of FDG for the assessment of sclerotic bone metastases of prostate carcinoma has also been noted [6]. So for primary staging, FDG is reported to be useful in selected patients with suspected high grade prostatic adenocarcinoma. Anatomic imaging modalities, which rely on size criteria, have also been reported to have low sensitivities for lymph node metastases in prostate cancer. Even in patients with high PSA levels (<25 ng/mL), the sensitivity of CT was found 30% to 35% [6]. Similarly, low sensitivity for nodal metastases (35%) was also reported in a summary of seven studies in which body magnetic resonance imaging was used for the staging of prostate cancer [7]. In our patient, metastatic right parailiac lymph nodes were well documented with FDG. Since there were no bone metastases, we could not evaluate the potential usage of FDG PET/CT in metastatic bone disease of squamous cell carcinoma of prostate. However, increased FDG uptake in sclerotic bone metastases has been reported in patients with breast carcinoma and it is postulated that it is not the type of metastases but the characteristics of the primary tumor that influence the degree of FDG uptake of bone metastases [8]. So it may be suggested that FDG avidity may be observed in bone metastases of squamous cell carcinoma of prostate.

In conclusion, squamous cell carcinoma of prostate should be considered in the differential diagnosis of abnormal FDG uptake of prostate. Apart from staging, in contrast to prostatic adenocarcinoma, FDG PET/CT may also play a role as a noninvasive test to direct biopsy for squamous cancer of prostate, as well.
